# Influence of sex, age and diabetes on brain transcriptome and proteome modifications following cerebral ischemia

**DOI:** 10.1186/s12868-023-00775-7

**Published:** 2023-01-27

**Authors:** Laura Ramiro, Júlia Faura, Alba Simats, Paula García-Rodríguez, Feifei Ma, Luna Martín, Francesc Canals, Anna Rosell, Joan Montaner

**Affiliations:** 1 Neurovascular Research Laboratory, Vall d’Hebron Institute of Research (VHIR), Universitat Autònoma de Barcelona, Barcelona, Spain; 2 Proteomics Laboratory, Vall d’Hebron Institute of Oncology (VHIO), Universitat Autònoma de Barcelona, Barcelona, Spain; 3 Stroke Research Program, Institute of Biomedicine of Seville, IBiS/Hospital Universitario Virgen del Rocío/CSIC/University of Seville & Department of Neurology, Hospital Universitario Virgen Macarena, Seville, Spain

**Keywords:** Ischemic stroke, Proteome, Transcriptome, Sex, Diabetes, Age

## Abstract

Ischemic stroke is a major cause of death and disability worldwide. Translation into the clinical setting of neuroprotective agents showing promising results in pre-clinical studies has systematically failed. One possible explanation is that the animal models used to test neuroprotectants do not properly represent the population affected by stroke, as most of the pre-clinical studies are performed in healthy young male mice. Therefore, we aimed to determine if the response to cerebral ischemia differed depending on age, sex and the presence of comorbidities. Thus, we explored proteomic and transcriptomic changes triggered during the hyperacute phase of cerebral ischemia (by transient intraluminal middle cerebral artery occlusion) in the brain of: (1) young male mice, (2) young female mice, (3) aged male mice and (4) diabetic young male mice. Moreover, we compared each group's proteomic and transcriptomic changes using an integrative enrichment pathways analysis to disclose key common and exclusive altered proteins, genes and pathways in the first stages of the disease. We found 61 differentially expressed genes (DEG) in male mice, 77 in females, 699 in diabetics and 24 in aged mice. Of these, only 14 were commonly dysregulated in all groups. The enrichment pathways analysis revealed that the inflammatory response was the biological process with more DEG in all groups, followed by hemopoiesis. Our findings indicate that the response to cerebral ischemia regarding proteomic and transcriptomic changes differs depending on sex, age and comorbidities, highlighting the importance of incorporating animals with different phenotypes in future stroke research studies.

**Supplementary Information:**

The online version contains supplementary material available at 10.1186/s12868-023-00775-7.

## Introduction

Ischemic stroke is a leading cause of death and disability worldwide [1]. Nowadays, recombinant tissue-plasminogen activator (rt-PA) and tenecteplase (TNK) are the only approved drug to treat acute ischemic stroke, although cerebral blood flow can also be restored by removing the obstructive clot through mechanical thrombectomy in selective patients [2, 3]. Despite the effectiveness of these reperfusion strategies, their short therapeutic window, some potential side effects and the strict clinical eligibility criteria impede offering these treatments to all stroke patients [4, 5]. Neuroprotective agents have been proposed as adjuvant therapies to be given during the hyperacute phase to minimize the impaired brain-damaged area while the ischemic brain is waiting for reperfusion therapy to restore the blood flow [6]. To date, more than 1,000 neuroprotective agents have shown promising results in preclinical models [7], but translation into the clinical setting has systematically failed [7–9].

The failure of the clinical trials with neuroprotective agents for the acute treatment of ischemic stroke makes the scientific community reconsider the most classic designs of preclinical studies. One of the reasons that could explain this failure in translation from “bench to bedside” is that the animal models used to test neuroprotectants do not represent properly the demographic and health characteristics of stroke patients [10]. Normally, pre-clinical studies include young animals, while it is known that more than 65% of all strokes occur in subjects older than 65 years [11] and only 10% in individuals under 50 years old [12, 13]. Regarding sex, most pre-clinical studies are performed on male animals, while stroke greatly impacts both sexes [14], being the first cause of death in women in some European countries [13, 15]. Finally, the vast majority of pre-clinical studies are performed in healthy animals, although stroke patients usually present a high incidence of comorbid conditions such as hypertension, diabetes and/or obesity, which are known to be among the most common stroke risk factors [16]. In this regard, the Stroke Therapy Academic Industry Roundtable (STAIR) published some recommendations to improve the quality of preclinical research and to make clinical implementation more efficient. Of note, these recommendations give special attention to species, strain and experimental model selection. In brief, they highlight the necessity of using both sexes in pre-clinical studies and highly recommend including animals with comorbidities and aged animals to better represent the human population that most likely will suffer stroke [17–19].

In this regard, it is plausible to think that the response to cerebral ischemia might be different depending on sex, age and comorbidities, and understanding these differences at the molecular level in the brain is fundamental to better comprehend stroke pathophysiology and to improve the “bench to bedside” translation. With this background, we aimed to explore proteomic and transcriptomic changes at a brain level triggered during the hyperacute phase of cerebral ischemia in mice of four different groups: (1) young male mice, (2) young female mice, (3) aged male mice and (4) diabetic young male mice. We aimed to compare the proteomic and the transcriptomic changes of each group using an integrative enrichment pathways analysis to determine if the response to ischemic stroke differed between the studied groups. Moreover, we aimed to reveal key common and exclusive dysregulated proteins, genes and pathways playing a crucial role in the first stages of the disease, with the hypothesis that those molecular changes could further determine the response to a given drug.

## Materials and methods

### Study design

The study presented here consists of a discovery study performed on RNA and protein extracts from mice ischemic brains of four different groups: (1) young male mice, (2) young female mice, (3) aged male mice and (4) diabetic young male mice, using mass spectrometry and RNA microarrays, with further integrative bioinformatics analysis. Each group included 8 ischemic animals, and 4 sham-control animals were used to discard genes and proteins differentially altered due to other phenomena rather than the ischemic event. Of note, the analysis of young male mice was performed in a first set of experiments [20]. The other 3 groups of animals were performed in a second set of experiments simultaneously.

### Animals

All animal procedures were performed in compliance with the Spanish legislation and in accordance with the Directives of the European Union and were approved by the Ethics Committee of the Vall d’Hebron Research Institute (protocol numbers 03/19 and 73/20). All experiments were conducted in a randomized manner and in adherence to the ARRIVE guidelines [21]. Group description: (1) C57BL/6 J male mice 8/12-week-old (referred as *males*), (2) C57BL/6 J female mice 8/12-week-old (referred as fe*males*), (3) C57BL/6 J male mice 18-month-old (referred as *old* [22]) and (4) BKS-Lepr^db/db^/JOrlRj diabetic male mice 8/12-week-old (referred as *diabetics*) (Janvier Labs, France). Animals were kept in a climate-controlled environment on a 12-h light/12-h dark cycle. Food and water were available ad libitum. Analgesia (Buprenorfine, 0.05 mg/kg, s.c, Divasa Farma-Vic S.A, Spain) was administered to all animals to minimize pain and discomfort. Anesthesia (isoflurane, 4% for induction, 2% for maintenance in medicinal air, Abbot Laboratories, Spain) was given via facemask during all surgical procedures described below.

A total of 57 animals were used for the present study. Among them, 9 were excluded after applying the following criteria: incomplete occlusion or reperfusion after removal of the filament (n = 2) and death during the experimental protocol (n = 7), while the other 48 were included.

### Transient middle cerebral artery occlusion (MCAO) model

Transient ischemia in the territory of the middle cerebral artery (MCA) was induced by introducing an intraluminal filament through the external carotid artery, as described before [23]. Briefly, animals were anesthetized and body temperature was maintained at 37 °C using a heating pad. The regional cerebral blood flow (CBF) was monitored close to the region irrigated by the MCA during the whole process by affixing a laser Doppler probe (Moor Instruments, UK) to the skull. Afterward, animals were placed in the supine position and the right bifurcation of the external carotid artery and internal carotid artery were exposed. Then, a silicone-coated nylon monofilament (Doccol Corporation, USA; 602256PK10for *males* and *females*, 702256PK5 for *diabetics* and 602456PK10 for *old* animals) was introduced through the external carotid artery to occlude the MCA. MCA occlusion (MCAO) was confirmed by a reduction in the cortical CBF recorded by the laser Doppler probe. Next, the incision was closed with a silk suture and animals were allowed to recover from anesthesia. Ninety minutes later, mice were re-anesthetized and the filament was removed to induce reperfusion of the MCA. After reperfusion, mice were allowed to recover from anesthesia under supervision. To ensure the correct occlusion of the MCA, only animals that exhibited a reduction of  ≥ 75% of CBF after filament introduction and a recovery of  ≥ 75% after filament removal were included in the study [24]. Sham animals underwent the same surgical procedures without the insertion of the nylon filament, and therefore, without MCAO.

### Brain collection and extraction of protein and RNA

Mice were deeply anesthetized (5% isoflurane) 30 min after reperfusion and transcardially perfused with 20 mL of cold saline. Immediately after perfusion, mouse brains were quickly removed and sectioned into 6 slices of 1 mm in cold conditions. The slice corresponding to the bregma anatomical point (representing the core of the infarct [25]) was carefully dissected to separate the right (ipsilateral, IP) and left (contralateral, CL) hemispheres separately. Each hemisphere was flash-frozen in liquid nitrogen and stored at − 80 °C.

Flash-frozen tissues were pulverized into powder in liquid nitrogen, and total fractions of protein and RNA were then isolated using the MirVana^™^ Paris^™^ kit (Thermo Fisher Scientific Inc., USA) following manufacturers’ instructions. RNA and protein fractions were stored at − 80ºC until further use.

### Transcriptomics study

Total RNA concentrations from brain samples were measured with a Nanodrop 1000 Spectrophotometer (ThermoFisher) and RNA integrity was assessed using the Agilent 2100 BioAnalyzer (Agilent Technologies, USA). Gene expression patterns were analyzed using Genechip© Mouse Clariom S 24 × arrays plates (Affymetrix, ThermoFisher). Starting material was 100 ng of total RNA of each sample. Briefly, sense ssDNA was generated from total RNA with the GeneChip WT Plus Reagent Kit (Affymetrix) following the manufacturer’s instructions. Then, sense ssDNA was fragmented, labeled and hybridized to the arrays with the GeneChip WT Terminal Labeling and Hybridization Kit (Affymetrix). Arrays plates were scanned and processed with Affymetrix GeneChip Command Console to obtain expression array intensity.cel files.

### Proteomics study

Due to equipment availability, the proteomics study was performed following two different protocols depending on the group of mice. Young male mice proteomic study was previously published [20]. Briefly, after in-gel digestion of the proteins, tryptic digests were analyzed using an LC–MS approach in a linear trap quadrupole (LTQ) Orbitrap Velos mass spectrometer (ThermoFisher).

For the remaining groups of animals, previous to LC-MS analysis, samples were digested with trypsin in-solution. Initial buffer exchange to 8 M Urea 50 mM ammonium bicarbonate (AB) was performed using 0.5 mL 3KDa cut-off Amicon Ultra ultrafiltration devices (Merck-Millipore). Total protein content was quantified using an RCDC kit (Bio-Rad), and 15 μg of each protein extract was taken for tryptic digestion. Samples were first reduced with DTT to a final concentration of 10 mM, for 1 h at room temperature (RT), and then alkylated with 20 mM of iodoacetamide (IAA) for 30 min at rt in the dark. Carbamidomethylation reaction was quenched by the addition of N-acetyl-L-cysteine to a final concentration of 35 mM followed by incubation for 15 min at RT in the dark. Samples were diluted with 50 mM AB to a final concentration of 1 M Urea, and then modified porcine trypsin (Promega Gold) was added in a ratio of 1:20 (w/w), and the mixture was incubated overnight at 37 °C. The reaction was stopped with formic acid (FA) to a final concentration of 0.5%. The tryptic digests were then purified on SCX spin columns (PolyLC), evaporated to dryness and stored at -20ºC until analyzed.

For LC-MS/MS analysis tryptic digests were diluted in 3% ACN, 1% FA and 500 ng of the sample was loaded to a 300 μm × 5 mm Pep-Map C18 (Thermo Scientific) at a flow rate of 15 μl/min using a Thermo Scientific Dionex Ultimate 3000 chromatographic system (Thermo Scientific). Peptides were separated using a C18 analytical column (NanoEase MZ HSS T3 column, 75 μm × 250 mm, 1.8 μm, 100 Å, Waters) with a 210 min run, comprising four consecutive steps, first 3 min of isocratic gradient at 3%B, from 3 to 35% B in 180 min, from 35 to 50% B in 5 min, from 50 to 85% B in 1 min, followed by isocratic elution at 85% B in 5 min and stabilization to initial conditions (A = 0.1% FA in water, B = 0.1% FA in CH3CN). The column outlet was directly connected to an Advion TriVersa NanoMate (Advion) fitted on an Orbitrap Fusion Lumos^™^ Tribrid (Thermo Scientific). The mass spectrometer was operated in data-dependent acquisition (DDA) mode. Survey MS scans were acquired in the orbitrap with the resolution (defined at 200 m/z) set to 120,000. The lock mass was user-defined at 445.12 m/z in each Orbitrap scan. The top-speed (most intense) ions per scan were fragmented in the linear ion trap (CID) and detected in the Ion Trap. Quadrupole isolation was employed to selectively isolate peptides of 350–1700 m/z. The predictive automatic gain control (pAGC) target was set to 1.5e5. The maximum injection time was set to 50 ms for MS1 and 35 ms for MS2 scan. Included charged states were 2–7. Target ions already selected for MS/MS were dynamically excluded for 30 s. The mass tolerance of this dynamic exclusion was set to ± 2.5 ppm from the calculated monoisotopic mass. The spray voltage in the NanoMate source was set to 1.7 kV. RF Lens were tuned to 30%. The minimal signal required to trigger MS to MS/MS switch was set to 5000 and activation Q was 0.250. The spectrometer was working in positive polarity mode and singly charge state precursors were rejected for fragmentation.

Progenesis_®_ QI for proteomics software v 3.0 (Nonlinear dynamics, UK) was used for MS data analysis using default settings. The results from LCMS runs were automatically aligned to a selected reference sample. Alignments were then manually supervised. Normalization of MS signals, based on the median of the ratiometric distribution of the abundance measurements, was performed automatically by the Progenesis^®^ QI for proteomics software. Only features within the 400 to 1600 m/z range, from 50 to 180 min of retention time, and with positive charges between 2 to 4 were considered for identification and quantification. Peaklists (mgf files) were generated using Progenesis^®^ QI for proteomics and searched from Proteome Discoverer 2.1 (Thermo Fisher Scientific Inc., USA) using the Mascot search engine (v5.1, Matrix Science, UK). Protein identification was carried out using the SwissProt-MusMusculus database (2020, Jul 01: 55,400 entries) setting precursor mass tolerance to 10 ppm and fragment mass tolerance to 0.5 Da. Oxidized methionine was considered as a variable amino acid modification and carbamidomethylation of cysteines as a fixed modification. Trypsin was selected as the enzyme allowing up to two missed cleavages. The significance threshold for the identifications was set to p < 0.05, minimum Mascot ions score of 20. Only those proteins quantified and identified with at least 2 unique peptides were considered for further statistical analyses.

### Bioinformatics and statistical analyses

Statistical and bioinformatics analyses of data were performed using custom scripts in R language (version 4.0.2) and R Studio (R Core Team, 2017, Vienna, Austria) with common *Bioconductor* packages.

As the male transcriptomic and proteomic studies were carried out in a first set of experiments separately from the other 3 groups, all groups have been analyzed independently.

#### Transcriptomics data

Transcriptomics data analyses have been performed separately for each group of animals. R olgo package was used to upload.cel files. Quality control of the data was performed using the *arrayQualityMetrics* package [26], a principal component analysis (PCA) and intensity boxplots for each file. Afterward, Robust Multiarray Average algorithm [27] was used for pre-processing microarray data to perform background adjustment, log2 transformation and quantile normalization. To obtain the expression measure of each probe, a linear model was fit to the normalized data. Next, genes whose standard deviation (SD) was below the 75 percentile of all SD were filtered out from the whole dataset.

The selection of differentially expressed genes was based on a linear model analysis with empirical Bayes modification for the variance estimates [28], using the *limma* package. This analysis has been performed considering sample pairing. False Discovery Rate (FDR)-based corrections for multiple testing were also calculated [29]. Fold-change (FC) was calculated dividing the IP by the CL expression value for each animal.

Sham animals’ data were used to discard genes differentially expressed due to other phenomena rather than cerebral ischemia. With this purpose, genes differentially expressed when comparing IP and CL both in sham and MCAO animals (FDR < 0.25) with a difference in logFC < 0.5 were discarded.

#### Proteomics data

Protein abundance quantification was based on the sum of the peak areas within the isotope boundaries of peptide ion peaks. Proteins identified by identical peptide sets were grouped to satisfy the principles of parsimony. Only those proteins quantified and identified with at least 2 unique and non-conflicting peptides (it is, features assigned unambiguously to peptides belonging to the protein, as assessed by Progenesis software). Protein abundance values were log-10 transformed and column-wise standardized [30]. Quality control of the data was performed using a principal component analysis (PCA) and intensity boxplots for each file.

The selection of differentially expressed proteins was based on a linear model analysis with empirical Bayes modification for the variance estimates [28], using the *limma* package. This analysis has been performed considering sample pairing. False Discovery Rate (FDR)-based corrections for multiple testing were also calculated [29]. Fold-change (FC) was calculated dividing the IP by the CL expression value for each animal.

#### Integrative analyses

An integrative analysis of the transcriptomics data of MCAO *male*, *female*, *old* and *diabetic* mice has been carried out. Venn diagrams were used to represent common and exclusive differentially expressed genes of studied groups. The common genes were used independently as input for STRING protein-protein interaction (PPI) network analysis. The interactions were based on text mining, experiments, databases, co-expression, neighborhood, gene fusion and co-occurrence, considering a medium confidence (0.4) [31].

For the integrative analysis, *ActivePathways* and its corresponding R package were used. In brief, this tool merges p-values and performs ranked hypergeometric tests to determine enriched pathways and processes [32]. First, a matrix was created with all the analyzed genes and their corresponding p-values for each dataset. Then, the p-values were merged using Brown’s method [33], obtaining a ranked gene list. Finally, ranked hypergeometric tests were carried out to determine if a pathway is enriched in the ranked gene list. The following functional gene sets were used: Gene Ontology (GO) Biological Processes and GO Molecular Functions [34, 35].

To visualize the enriched pathways as a network, Cytoscape software [36] and the EnrichmentMap app [37] were used.

## Results

### Transcriptomics results

We determined changes in gene expression that occur in the brain within the hyperacute phase after ischemic stroke by analyzing mouse-brain samples obtained 2 h after cerebral ischemia induction (30 min after reperfusion) or sham surgery. The changes in gene expression were elucidated using microarrays, comparing the IP and CL regions and correcting by sham animals for each studied group.

Quality control of samples was performed for each group of animals. Regarding *diabetic*, *female* and *old* mice all samples passed the quality control. However, after performing quality control in *male* mice, 2 ischemic animals (4 samples) were clearly separated from the rest in PCA with lower intensities than the other individuals, which remained after normalization. For that reason, these 2 animals were removed from the analysis. Afterward, all samples passed the quality control. So, for the transcriptomics study young female mice, aged male mice and diabetic young male mice groups included 8 ischemic animals and 4 sham-control animals, while the young male mice group included 6 ischemic animals and 4 sham-control animals.

#### Differentially expressed genes

In *male* mice, we found 61 differentially expressed genes (DEG) after MCAO when comparing the IP and the CL brain regions (FDR < 0.25), while in sham-control animals we did not identify any DEG. In *female* mice, there were 77 DEG between IP and CL brain regions in MCAO animals (FDR < 0.25), while only 8 DEG were found in sham animals (FDR < 0.25) and there were no common dysregulated genes between *female* MCAO and sham animals. In *diabetic* mice, 699 DEG were found when comparing the IP and the CL brain regions in MCAO animals (FDR < 0.25), while in sham control animals there were no DEG. Finally, in *old* mice we found 24 DEG were found when comparing the IP and the CL brain regions in MCAO animals (FDR < 0.25), while in sham control animals there were no DEG. Details of the top 15 DEG after cerebral ischemia of each studied group are shown in Table 1.

**Table 1:** Top 15 differentially expressed genes (FDR < 0.25) between the infarcted hemisphere and the contralateral healthy hemisphere 2 h after cerebral ischemia induction. Genes that are differentially expressed in all the groups of animals are highlighted in bold. FDR: false discovery rate; logFC: Logarithmic fold change

Young male mice, *males*				Young female mice, *females*				Diabetic young male mice, *diabetic*				Aged male mice, *old*			
SYMBOL	logFC	P.Value	FDR	SYMBOL	logFC	P.Value	FDR	SYMBOL	logFC	P.Value	FDR	SYMBOL	logFC	P.Value	FDR
*CCL3*	2.4958	1.73E-15	8.80E-12	*ATF3*	1.4557	1.42E-06	0.00284	*HSPA1A*	2.4544	1.02E-09	5.17E-06	*NPAS4*	2.9274	1.53E-08	7.75E-05
*FOSB*	1.9774	7.77E-12	1.58E-08	*PTGS2*	1.5976	1.76E-06	0.00284	*FOS*	2.4266	9.29E-09	1.63E-05	*CCL3*	2.0499	1.08E-07	2.74E-04
*CCN1*	1.8493	1.10E-11	1.58E-08	*CCRL2*	1.1296	2.21E-06	0.00284	*CCN1*	1.9876	9.61E-09	1.63E-05	*FOS*	1.6891	8.48E-07	0.00143
*FOS*	2.1172	1.25E-11	1.58E-08	*FOS*	1.5602	2.24E-06	0.00284	*CCL3*	2.6185	1.55E-08	1.97E-05	*IL1A*	1.7778	2.60E-06	0.00329
*NPAS4*	2.6951	7.08E-11	7.18E-08	*CCL3*	2.1777	2.93E-06	0.00298	*FOSB*	2.1383	4.27E-08	4.33E-05	*PTGS2*	1.3054	5.90E-06	0.00599
*ATF3*	1.6062	1.09E-09	9.22E-07	*NPAS4*	2.2737	4.40E-06	0.00372	*GADD45G*	1.7414	5.81E-08	4.91E-05	*FOSB*	1.5304	9.64E-06	0.00815
*JUNB*	1.4066	2.36E-09	1.67E-06	*ADAMTS1*	1.0314	6.65E-06	0.00482	*IER2*	1.2020	9.91E-08	7.18E-05	*DUSP6*	0.9922	3.29E-05	0.02382
*RGS2*	1.4974	2.80E-09	1.67E-06	*CCN1*	1.3156	9.48E-06	0.00601	*THBS1*	1.8039	1.14E-07	7.22E-05	*HSPA1A*	1.6433	7.39E-05	0.04390
*GADD45G*	1.3902	2.96E-09	1.67E-06	*NR4A1*	1.0643	1.54E-05	0.00868	*NPAS4*	3.1196	3.98E-07	2.25E-04	*JUN*	0.8117	8.45E-05	0.04390
*HSPA1A*	1.5433	3.32E-09	1.68E-06	*FOSB*	1.5493	1.88E-05	0.00951	*DUSP6*	1.1689	5.27E-07	2.67E-04	*CCL4*	1.1057	8.66E-05	0.04390
*EGR4*	1.4966	8.59E-09	3.79E-06	*RND3*	0.8880	2.34E-05	0.01079	*NR4A1*	1.6955	7.58E-07	3.49E-04	*FKBP11*	-1.0158	1.84E-04	0.08205
*NR4A1*	1.4166	8.97E-09	3.79E-06	*RGS1*	1.1871	2.88E-05	0.01216	*EGR4*	1.6425	9.33E-07	3.94E-04	*GM9958*	-0.9378	2.12E-04	0.08205
*PTGS2*	1.3968	1.51E-08	5.88E-06	*GADD45G*	1.0496	3.97E-05	0.01547	*RGS2*	1.3091	1.09E-06	4.23E-04	*NR4A1*	1.1258	2.21E-04	0.08205
*BTG2*	1.2922	2.79E-08	1.01E-05	*DUSP6*	0.8763	4.39E-05	0.01547	*JUN*	1.1095	1.17E-06	4.23E-04	*CCRL2*	0.9681	2.27E-04	0.08205
*EGR2*	1.3976	4.45E-08	1.51E-05	*GALNT12*	-1.1040	4.58E-05	0.01547	*AKAP12*	0.9770	1.51E-06	5.10E-04	*CCN1*	1.2480	2.56E-04	0.08651

#### Integrative analysis of transcriptomic results

**Fig. 1:**
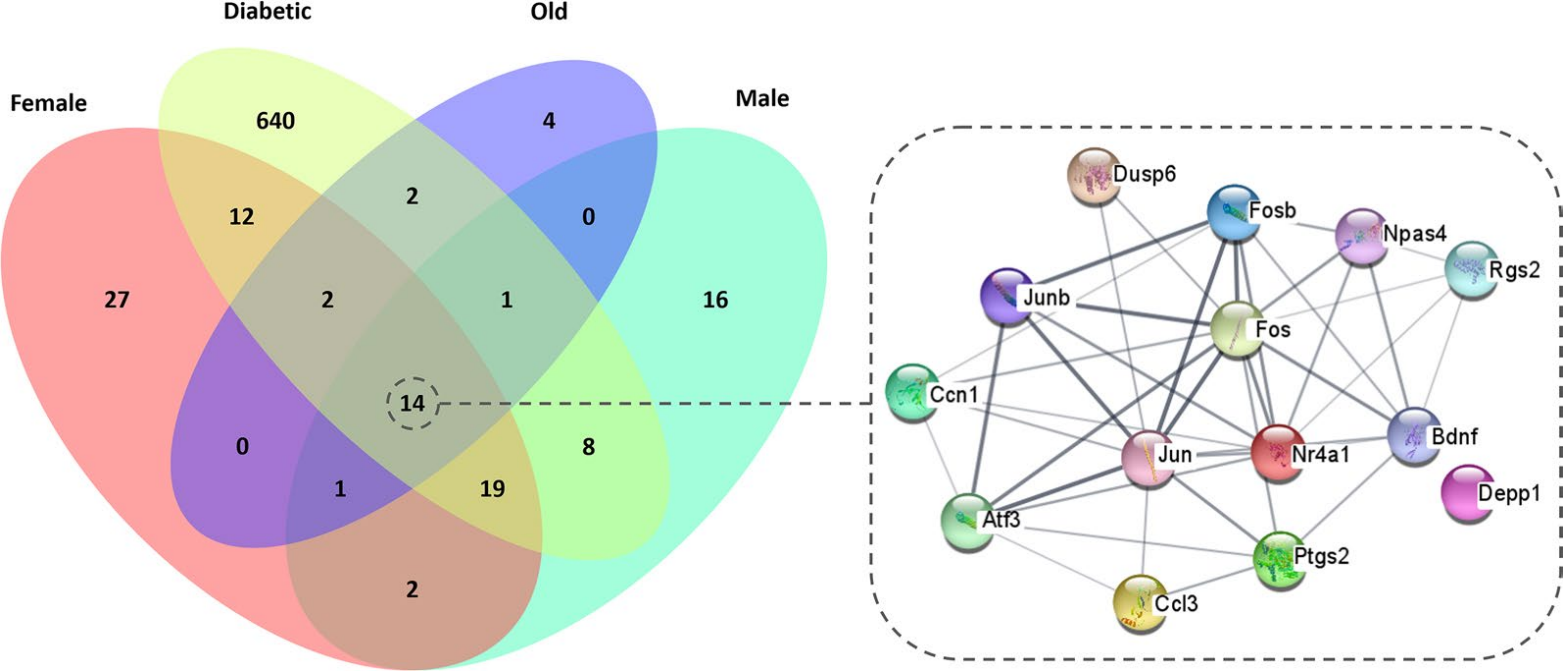
Venn diagram of differentially expressed genes in the ischemic brain (FDR < 0.25) in all studied groups. The 14 common differentially expressed genes are shown through a STRING network revealing the main interactions between molecules. The strength of the correlation between two genes (nodes) 20 is reflected by the thickness of the edge. Diabetic: diabetic young male mice; Female: young female mice; Male: young male mice; Old: aged male mice

To understand the mechanisms underlying ischemic stroke, we sought to identify common DEG after cerebral ischemia in all groups, as they might be playing a crucial role in ischemic stroke response and thus be pointing at crucial therapeutic targets in the acute phase of the disease. We found 14 DEG in the ischemic brain that were common in all four groups of animals (IP vs CL, FDR < 0.25). In addition, it is worth noting that 27 genes were dysregulated only in *female* mice, 640 in *diabetic* mice, 4 in *old* mice and 16 in *male* mice (Fig. 1, Additional file 1: Table S1).

**Fig. 2:**
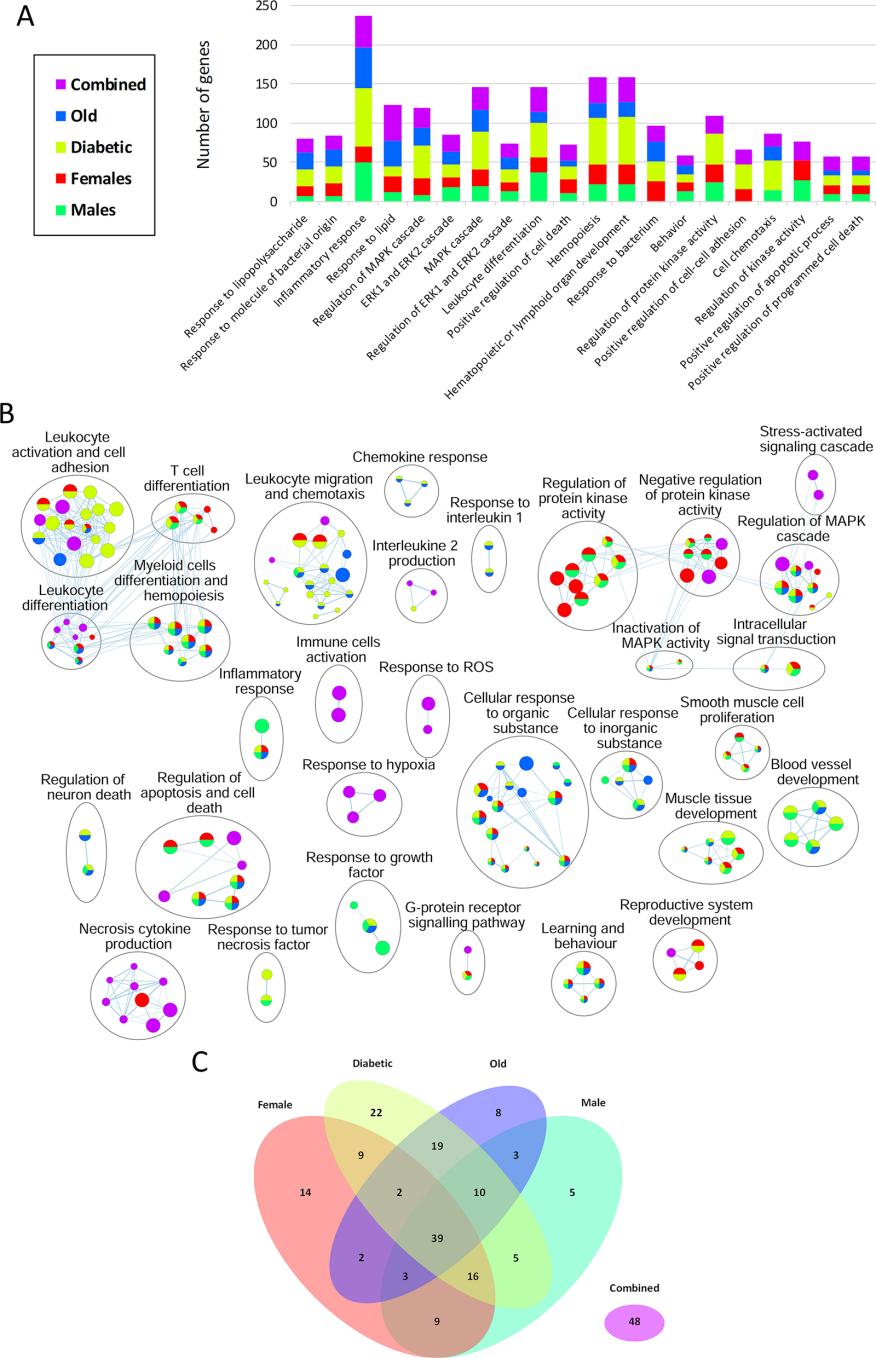
Pathway enrichment analysis revealing the main biological processes of differentially expressed genes in the brain 2 h after cerebral ischemia. **A** Top 20 enriched biological processes; bar plot showing the number of differentially expressed genes of each altered biological process, sorted by increasing p-value from left to right. Each color indicates the corresponding transcriptomic dataset, and the purple color (integromics) corresponds to a subset of enriched pathways with combined evidence that were only detected by integrating the data of all groups. **B** Enrichment map of biological processes altered acutely after ischemic stroke. Nodes in the network represent biological processes, and similar biological processes with many common genes are connected. Nodes are colored according to the supporting omics datasets. Only pathways with 2 or more nodes were represented. **C** Venn diagram of the enriched biological processes in all studied groups. Diabetic: diabetic young male mice; Female: young female mice; Male: young male mice; Old: aged male mice

**Fig. 3:**
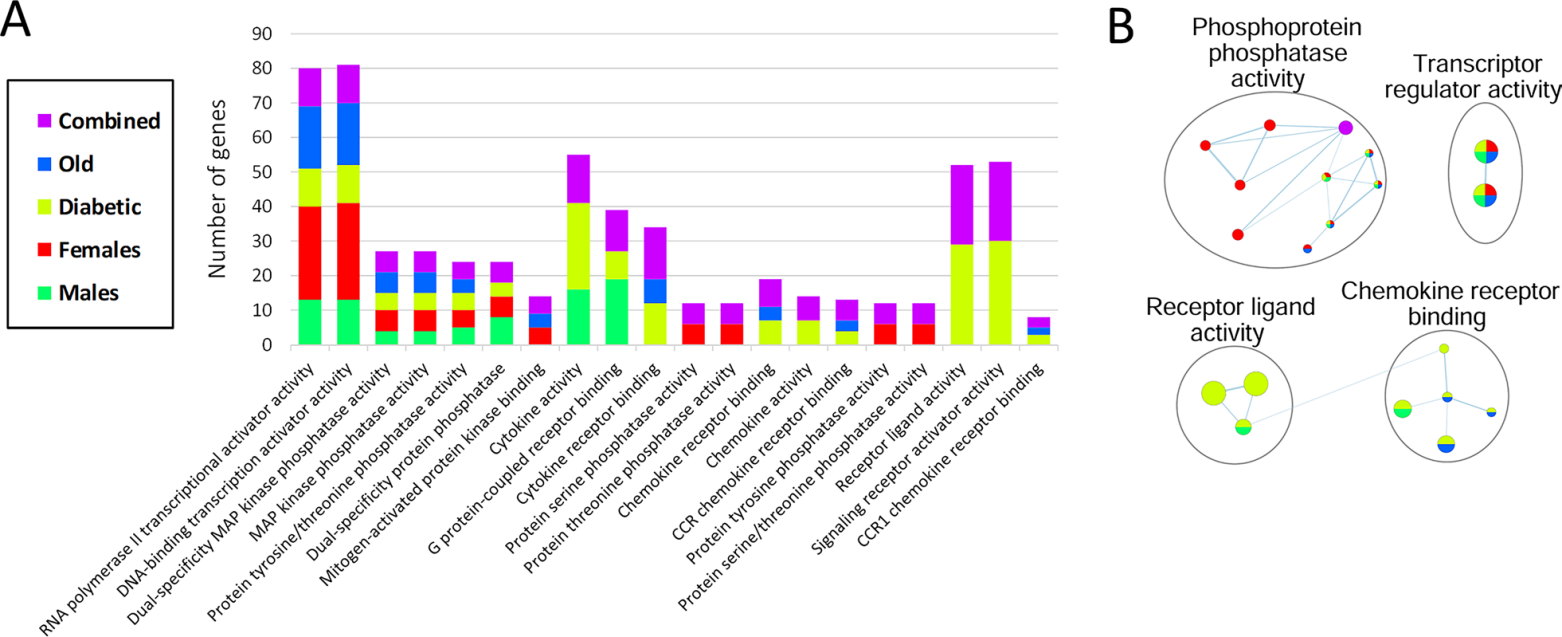
Pathway enrichment analysis revealing the main molecular functions of differentially expressed genes in the brain 2 h after cerebral ischemia. **A** Top 20 enriched molecular functions; bar plot showing the number of differentially expressed genes of each altered molecular function, sorted by increasing p-value from left to right. Each color indicates the corresponding transcriptomic dataset, and the purple color (integromics) corresponds to a subset of enriched pathways with combined evidence that were only detected by integrating the data of all groups. **B** Enrichment map of molecular functions altered after ischemic stroke. Nodes in the network represent molecular functions and similar molecular functions with many common genes are connected. Nodes are colored according to the supporting omics datasets. Diabetic: diabetic young male mice; Female: young female mice; Male: young male mice; Old: aged male mice

The multi-omics pathway enrichment analysis was conducted on the integrated gene data to further understand transcriptomic alterations triggered by cerebral ischemia. Interestingly, this analysis pointed out enriched pathways on every single dataset but also enriched pathways that are only found through data integration that are not apparent in any single omics dataset independently [32]. This analysis identified several biological processes whose genes are significantly enriched within the hyperacute phase of cerebral ischemia. The top 20 enriched biological processes (sorted by p-value) and the number of DEG for each group are shown in Fig. 2A. We created an enrichment map that shows pathways whose genes are significantly enriched in transcriptomics datasets. Remarkably, the acute inflammatory response was the biological process that encompassed more DEG, followed by hematopoietic or lymphoid organ development, hemopoiesis, MAPK cascade and leukocyte differentiation. Moreover, the enrichment map of biological processes revealed 478 genes that were significantly enriched in 214 biological processes (Fig. 2B). Of these 214 biological processes, 39 were common in all groups, 14 were only dysregulated in *females*, 22 in *diabetics*, 8 in *olds*, 5 in *males* and 48 were found only in the integrated gene list of all groups (Fig. 2C). Furthermore, the main molecular functions of these DEG were related to phosphoprotein phosphatase activity, chemokine receptor binding, receptor ligand activity and transcription regulator activity, being this last one the molecular function with more DEG, especially in females (Fig. 3A–B).

### Proteomics results

We also analyzed protein changes occurring early after cerebral ischemia in the brain using mass spectrometry. All samples in each group of animals passed the quality control. So, each group of animals included 8 ischemic animals and 4 sham-control animals.

After MCAO induction, 4 proteins were found to be differentially expressed between the IP and the CL brain regions in male mice (FDR < 0.25), 14 in *female* mice and 799 in *diabetic* mice. However, when comparing both regions, there were no differentially expressed (FDR < 0.25) proteins in *old* mice. Moreover, there were no proteins differentially expressed (FDR < 0.25) between the IP and the CL in sham animals of any of the groups. The top 15 proteins differentially expressed after cerebral ischemia for each group are shown in Table 2.

**Table 2:** Top 15 proteins differentially expressed (FDR < 0.25) between the ipsilateral hemisphere and the contralateral hemisphere 2 h after cerebral ischemia induction FDR false discovery rate; logFC Logarithmic fold change

Young male mice, *males*				Young female mice, *females*				Diabetic young male mice, *diabetic*				Aged male mice, *old*			
UNIPROT	logFC	P.Value	FDR	UNIPROT	logFC	P.Value	FDR	UNIPROT	logFC	P.Value	FDR	UNIPROT	logFC	P.Value	FDR
Q8CI32/Bag5	− 0.3250	2.82E-08	7.01E-05	Q8BKX1/Baiap2	0.4799	1.62E-05	0.0413	Q8VBY2/Camkk1	− 0.4021	1.55E-07	3.85E-04				
P11798/Camk2a	− 0.2119	9.70E-05	0.1205	Q8CGY8/Ogt	− 0.2743	1.29E-04	0.1068	O70433/Fhl2	− 0.6287	5.24E-07	6.51E-04				
Q6PHZ2/Camk2d	− 0.1114	3.09E-04	0.2427	Q920P5/Ak5	− 0.2659	1.43E-04	0.1068	Q62318/Trim28	− 0.2786	1.21E-06	0.0010				
O35927/Ctnnd2	− 0.0972	3.91E-04	0.2427	F6SEU4/Syngap1	0.4880	1.93E-04	0.1068	Q8R570/Snap47	− 0.4342	6.25E-06	0.0037				
				P68404/Prkcb	− 0.2485	2.09E-04	0.1068	P27005/S100a8	0.7479	7.42E-06	0.0037				
				P08414/Camk4	− 0.3514	4.61E-04	0.1680	P08414/Camk4	− 0.5602	9.31E-06	0.0039				
				Q9Z2Y3/Homer1	0.2486	5.01E-04	0.1680	Q64337/Sqtm1	− 0.4070	1.33E-05	0.0041				
				E9Q0K9/Elmod1	− 0.3820	5.27E-04	0.1680	Q9WV34/Mpp2	− 0.2949	1.34E-05	0.0041				
				Q8BFT9/Svop	0.2494	7.22E-04	0.2046	Q8BFR5/Tufm	− 0.3072	1.68E-05	0.0046				
				Q61329/Zfhx3	0.1805	8.62E-04	0.2200	Q8K0E8/Fgb	0.8275	1.99E-05	0.0049				
				Q8VEA4/Chchd4	0.2649	1.01E-03	0.2201	Q3UHD6/Snx27	− 0.5525	2.30E-05	0.0049				
				E9QK62/Ngef	− 0.2693	1.04E-03	0.2201	Q68FF6/Git1	− 0.4539	2.38E-05	0.0049				
				P97822/Anp32e	− 0.1824	1.19E-03	0.2332	Q920P5/Ak5	− 0.5258	2.73E-05	0.0052				
				O88737/Bsn	0.2131	1.28E-03	0.2332	P68404/Prkcb	− 0.2960	3.87E-05	0.0069				
								P04627/Araf	− 0.4653	4.54E-05	0.0070				

#### Integrative analysis of proteomic results

We aimed to identify common proteins dysregulated in the ischemic brain after stroke in all groups, as they will be playing a key role in stroke pathophysiology. However, we found no proteins differentially expressed shared between all the groups. Interestingly, females and diabetic mice were the only groups sharing dysregulated proteins, with 10 common proteins.

## Discussion

The present study identifies key transcriptomic and proteomic changes triggered acutely after cerebral ischemia in brain samples of mice of different groups: (1) young male mice, (2) young female mice, (3) aged male mice and (4) diabetic young male mice, through mass spectrometry and microarrays. Moreover, the transcriptomics data generated has been integrated and analyzed together to reveal molecules and pathways with key roles in the hyperacute phase of stroke pathophysiology.

To date, various studies have devoted their efforts to describe proteomic and transcriptomic changes that occur in the brain after stroke using mice models of cerebral ischemia. These studies have provided valuable knowledge that has contributed to a better characterization of stroke pathology. This better comprehension of the disease has aided in selecting new candidates to be studied as neuroprotectants and/or biomarkers [38–40]. However, the vast majority of these studies are performed in young healthy male mice, with relatively fewer studies using females, and anecdotic studies using mice with comorbidities and aged mice [10]. However, it has been demonstrated that the response triggered after a stroke is affected by sex, age and comorbidities [41, 42]. Importantly, it has been suggested that this lack of precision in representing the population mostly affected by stroke in pre-clinical studies, might be one of the reasons explaining the systematic failure in translating the promising neuroprotection results obtained in pre-clinical studies into the clinical setting [10, 43]. Taking all this into account, we aimed at evaluating similarities and differences in protein and gene changes triggered acutely after cerebral ischemia in mice of four different groups differing in sex, age and the presence of comorbidities to elucidate if these differences could explain the off-target molecules of choice when using standard young male models.

Regarding the transcriptomics results, it is worth noting that the number of DEG after cerebral ischemia is similar between *males* and *females* (61 and 77 respectively), while in *old* animals the number of DEG is reduced to 24 and in *diabetic* animals there is an important increase up to 699. In this line, our results suggest that the post-ischemic expression level of transcripts might be attenuated with age. In fact, an age-dependent decline in transcriptional homeostasis has been described before and is being extensively studied, although to date is poorly understood [44, 45]. On the contrary, the expression level of transcripts after cerebral ischemia could be exacerbated by the presence of comorbidities such as diabetes, indicating that cerebral ischemia might be altering a higher number of molecular processes and pathways in that latter group of animals.

Considering the top 15 genes that are differentially expressed in each group (regarding their FDR), most of them are common in all four groups and mainly encode for transcription factors, chemokines and GTPases, which are known to be key players during the hyperacute phase of cerebral ischemia [46–49]. For example, NPAS4 encodes for a transcription factor whose expression is induced in various brain insults, including cerebral ischemia [50, 51]. NPAS4 indeed has a neuroprotective role in cerebral ischemia, probably being involved in cell death and inflammatory response modulation [52]. In addition, CCL3 is an active mediator in cerebral post-ischemic inflammation, upregulated in the ischemic core [53], crucial for the induction of monocyte and neutrophil recruitment at inflammatory sites [54]. Moreover, upregulation of ATF3 transcript in the ischemic core during the hyper-acute phase has previously been described in young male mice [55]. In fact, this study revealed that ATF3 upregulation might be related to mechanisms underlying mitigation of the neurotoxicity at an early stage after ischemia. In the same line, it has been shown that NR4A1 mRNA expression is rapidly induced in the ischemic core in a rat model of focal cerebral ischemia [56, 57]. Besides, it is thought that NR4A1 plays an important role in the regulation of neuroinflammation and in neural apoptosis after ischemic stroke [58, 59].

From all the DEG, it is worth noting that 35 of them are shared between *males* and *females*, but 27 are exclusive for females and 16 for males, highlighting that divergences due to sex are an important factor to be considered. In fact, the existence of significant sexual dimorphism in stroke response has been described before. For example, genetic and epigenetic factors, differential activation of cell-death programs, cell-cell signaling pathways, and systemic immune responses have been proposed as contributors to sex differences in ischemic stroke [41]. Actually, various cell signaling pathways such as ischemia-induced cell death, mitochondrial metabolism and inflammation are different between males and females [60–62].

In reference to *old* mice, our results show that there is a significant reduction in the number of DEG after the insult, being most of the DEG shared with the other groups and only 4 of them exclusively dysregulated in *old* mice. Interestingly, a previous study exploring the effect of age on the transcriptional response to ischemic stroke, reported that aged mice differentially regulated more genes than young mice 3 days after the event [63]. In the present study, we have found that within the hyperacute phase of the disease (2 h after the occlusion) aged animals have less DEG than their young counterparts, which might suggest that the transcriptional response to cerebral ischemia might be a slower process in aged mice, although at later time points the response might be exacerbated. Regarding the biological processes and molecular functions altered after ischemic stroke, we found clear differences between aged mice and the other groups regarding cell adhesion processes and regulation of protein kinase activity, which seem to be unaltered in aged animals. Protein kinases are involved in the majority of cellular pathways, especially cell signaling and signal transduction [64, 65]. In addition, cell adhesion processes are triggered after cerebral ischemia to mediate the infiltration of leukocytes into the brain parenchyma [66]. The fact that we did not find significant DEG involved in these two processes in aged mice, highlights the importance of further studying their relationship with aging and stroke response. In this regard, considering that aged patients have poorer outcomes and higher rates of mortality after stroke, pharmacologically modulating molecules involved in these biological processes during the hyperacute phase of the disease in the aged population might be an interesting approach to be evaluated in the future as neuroprotective strategies [67].

The presence of comorbidities such as obesity, diabetes and hypertension are known risk factors for ischemic stroke [16]. Moreover, patients with these comorbidities have poorer outcomes after cerebral ischemia [68–70]. We found that diabetic mice have a significant increase in the number of DEG after stroke, being 10 times higher than in the other groups, which might suggest that cerebral ischemia triggers the activation and modulation of an augmented number of biological processes in that group of animals. It has been reported before that after cerebral ischemia diabetic mice had pronounced systemic and vascular inflammation, augmented blood-brain barrier disruption, increased pro-inflammatory response, metabolic dysregulation, severe brain damage and worse neurological deficits [71–73]. Our results support this idea, given that the number of DEG in diabetic mice related to these biological processes is significantly higher than in the other groups. Interestingly, we found an increased number of DEG related to cell adhesion and leukocyte activation that were exclusively dysregulated in diabetic mice. In fact, it has been described that diabetic mice had an augmented number of infiltrating leukocytes in the brain after stroke, indicating that these cells might be responsible for the exacerbated ischemic brain injury in this group of animals [73].

The enrichment pathways analysis revealed that the inflammatory response and hemopoiesis were key biological processes involved in the animal differences. The inflammatory response triggered after the ischemic event plays an important role in the progression of stroke. It has been described that it has a dual role, as it exerts deleterious effects but also contributes to brain protection and repair of the damaged area [74–76]. Our results show that *males* had a greater number of DEG related to this process than *females*. It has been shown that sex hormones can regulate the immune system, as the majority of immune cells have receptors for these molecules [77]. In the same line, progesterone has been associated with a reduction of pro-inflammatory cytokines, while depending on the context estrogen can either promote or inhibit the inflammatory response [78–80]. Moreover, diabetes and aging exacerbate inflammatory mechanisms, increasing the susceptibility of the blood-brain barrier to damage, consequently augmenting the risk of hemorrhagic transformation, reperfusion injury, and cerebral edema following stroke, and also countering endogenous resilience and recovery mechanisms activated after stroke [81, 82]. In this regard, there are various neuroprotective agents targeting inflammation that have shown promising results in pre-clinical studies but have failed in clinical translation, such as Anakinra, Enlimomab and Natalizumab [83]. Considering our results, it is plausible to think that the differences observed in the regulation of inflammatory response might be one of the possible reasons influencing the failure of translating the preliminary results obtained into the whole population.

Regarding hemopoiesis, various studies have revealed that acute cardiovascular events like myocardial infarction or stroke greatly impact hemopoiesis via sympathetic activation, glucocorticoid release, and the production of various proinflammatory molecules [84–86]. Indeed, the systemic number of innate immune cells acutely increases after stroke. It has been demonstrated that bone marrow hematopoietic stem cells (HSC) and downstream hemopoietic progenitors augment their activity after MCAO in mice, leading to an increased output of inflammatory monocytes and neutrophils [87]. However, it has been described that common cardiovascular risk factors such as arterial hypertension, hyperlipoproteinemia, and diabetes mellitus considerably alter hemopoietic processes [86]. In the same line, it has been demonstrated that age also influences hematopoiesis, given that stem cells become more poorly functional and there is a reduction in T-cell production, which may contribute to a decrease in immunity seen in the elderly [88]. Regarding sex differences, various studies revealed that sex hormones exert significant effects on hemopoiesis through selectively expressed receptors in various populations of stem/progenitor cells, controlling their self-renewal, differentiation, and proliferation [89]. Thus, considering that the inflammatory response and the hemopoiesis are key processes triggered after stroke, the differences described in these mechanisms depending on phenotypes might be ultimately influencing the stroke response, explaining the differences observed in the present study.

The proteomics results are in agreement with the ones found in transcriptomics. Diabetic animals had an enormous number of proteins altered in the ischemic core in comparison to all other groups, while in *old* animals we did not find any dysregulated protein, maybe due to the early time-point in which samples were obtained. Regarding *males* and *females*, we found a similar number of altered proteins in both groups, although it was higher in females, as observed in the transcriptomics results. In general, our results suggest that in the studied time-point genes had a higher contribution to the observed alterations triggered after cerebral ischemia than proteins. One possible reason for this might be the early time point (2 h after occlusion) in which we evaluated the changes triggered after ischemia. In this regard, performing a similar study and analysis at later time points will give complementary and valuable information to better characterize divergences and similarities in cerebral ischemia response between the studied groups. In addition to this, various biological reasons could be influencing the observed divergence between altered proteins and genes such as differential post-transcriptional mechanisms, the tight regulation of translation, and differences in the turnover of proteins and mRNAs [90–92]. Given the scarce amount of dysregulated proteins that we obtained in 3 of the studied groups, we were not able to perform an integrative analysis.

Altogether, we show robust data supporting differences in the molecular responses triggered by cerebral ischemia depending on the sex, age and the presence of comorbidities. These divergences might be one of the reasons explaining the choice of inappropriate therapeutic targets and the posterior failure in clinical translation of the main neuroprotective agents tested in pre-clinical studies up to the date. To improve future study designs to make clinical implementation more effective, the Stroke Therapy Academic Industry Roundtable (STAIR) published some recommendations to enhance the quality of preclinical research. Remarkably, these recommendations give special attention to the experimental model, species and strain selection and emphasize the necessity of considering both sexes when designing experiments. In addition, they highly advocate including animals with comorbidities such as diabetes, obesity or hypertension as well as aged animals in pre-clinical studies, to better represent the human population that most likely will suffer a stroke [17–19]. The results obtained in the present study robustly support the necessity of implementing these recommendations when designing pre-clinical studies to test neuroprotective agents. In this regard, molecules to be targeted in pre-clinical studies should be selected considering the differences and similarities that exist between groups. Moreover, the presence of molecules exclusively dysregulated in one group of animals might open the door to future studies directed towards personalized medicine in subcohorts of stroke patients. For example, if the functional analysis of molecules that are dysregulated in diabetic, *males* and *females* but not in *old* mice showed neuroprotective effects, a pharmacologic activation of these molecules in aged ischemic brains might improve the functional outcome in elderly stroke patients. Conversely, if one of the many molecules dysregulated in diabetic mice shows negative neurodegenerative or even neurotoxic functions, a pharmacological inhibition in diabetic ischemic brains could bear neuroprotective effects. This approach will definitely provide a personalized management of stroke patients, by treating each patient considering its clinical characteristics.

This study stands for some limitations that had to be considered. First, regarding the experimental design, ideally, young male mice should have been performed simultaneously with the other groups, to reduce inter-assay variability. In this line, to minimize the variability, each group of animals was analyzed separately and corrected by its own control samples. Second, in the present study, we have incorporated 4 different groups of animals differing in sex, age or the presence of diabetes. However, it would have been also interesting to include additional groups such as aged females, diabetic females or mice with other comorbidities such as hypertension to enrich the obtained information. Third, time extrapolations between mice and humans are poorly understood and not well established. A better comprehension of this equivalence is needed to better comprehend the implications of the results presented here. Moreover, the early time-point in which we have evaluated the changes triggered after ischemic stroke seems not to be the optimum to find proteomic divergences between groups. Finally, infarct volume was not assessed in the present study due to the lack of sensitivity of TTC (triphenyltetrazolium chloride) staining to determine the lesion volume 2 h after occlusion.

All in all, in the present study we have explored similarities and differences in protein and gene changes in the brain after cerebral ischemia depending on age, sex and the presence of diabetes. In this regard, we have revealed that the molecular response to stroke varies depending on the phenotype of the animals, reinforcing the necessity of following STAIR recommendations when designing future studies to improve “bench to bedside” translation.

## Supplementary Information

**Additional file 1: Table S1.** Top 30 differentially expressed genes (FDR<0.25) between the infarcted hemisphere and the contralateral healthy hemisphere 2 hours after cerebral ischemia induction exclusive from each group of animals.

## Data Availability

The mass spectrometry proteomics data have been deposited to the ProteomeXchange Consortium via the PRIDE [93, 94] partner repository with the dataset identifier PXD032141, and the transcriptomics data in the Gene Expression Omnibus (GEO) database with the dataset identifier GSE196266.
