# Supplementary material for: Influence of sex, age and diabetes on brain transcriptome and proteome modifications following cerebral ischemia
Source: BMC Neurosci. 2023 Jan 27;24:7. doi: 10.1186/s12868-023-00775-7 (PMC9881265; doi:10.1186/s12868-023-00775-7)
Supplement: Supplementary file 1 — Additional file 1: Table S1. Top 30 differentially expressed genes (FDR<0.25) between the infarcted hemisphere and the contralateral healthy hemisphere 2 hours after cerebral ischemia induction exclusive from each group of animals. [file 12868_2023_775_MOESM1_ESM.docx]

**Supplementary material**

**Supplemental table S1.** Top 30 differentially expressed genes (FDR<0.25) between the infarcted hemisphere and the contralateral healthy hemisphere 2 hours after cerebral ischemia induction exclusive from each group of animals. FDR: false discovery rate; logFC: Logarithmic fold change.

| **Young male mice** | | | | **Young female mice** | | | | **Diabetic young male mice** | | | | **Aged male mice** | | | |
| --- | --- | --- | --- | --- | --- | --- | --- | --- | --- | --- | --- | --- | --- | --- | --- |
| **SYMBOL** | **logFC** | **P.Value** | **FDR** | **SYMBOL** | **logFC** | **P.Value** | **FDR** | **SYMBOL** | **logFC** | **P.Value** | **FDR** | **SYMBOL** | **logFC** | **P.Value** | **FDR** |
| Cldn20 | -0.7665 | 1.84E-04 | 0.0291 | Galnt12 | -1.1040 | 4.58E-05 | 0.0155 | S100a9 | 1.4113 | 2.29E-06 | 6.44E-04 | Fkbp11 | -1.0158 | 1.84E-04 | 0.0820 |
| Themis2 | 0.7839 | 1.97E-04 | 0.0302 | Olfr690 | -1.0280 | 4.99E-05 | 0.0158 | S100a8 | 1.5050 | 5.19E-06 | 1.14E-03 | Gm9958 | -0.9378 | 2.12E-04 | 0.0820 |
| Dgat2l6 | -0.6872 | 3.30E-04 | 0.0468 | Bcl2a1c | 0.8490 | 1.09E-03 | 0.1478 | Ccl7 | 1.1113 | 1.18E-05 | 2.22E-03 | Dnajb13 | 0.6565 | 1.14E-03 | 0.2467 |
| Gata2 | -0.6329 | 9.20E-04 | 0.1166 | Foxf1 | 0.7005 | 1.12E-03 | 0.1478 | Pstk | -0.9176 | 1.58E-05 | 2.77E-03 | Krtap12-1 | -0.7606 | 1.17E-03 | 0.2467 |
| Asb5 | -0.6768 | 1.02E-03 | 0.1245 | Tnfrsf10b | 0.8084 | 1.14E-03 | 0.1478 | Ccl2 | 0.9432 | 2.94E-05 | 4.25E-03 |  |  |  |  |
| Bmp15 | 0.6382 | 1.03E-03 | 0.1245 | Mmp10 | -0.8206 | 1.25E-03 | 0.1520 | Creb5 | 0.7937 | 3.27E-05 | 4.61E-03 |  |  |  |  |
| Cited2 | 0.6148 | 1.20E-03 | 0.1385 | Olfr492 | 0.9696 | 1.32E-03 | 0.1524 | Hp | 0.9923 | 3.90E-05 | 5.20E-03 |  |  |  |  |
| Ccl19 | -0.6130 | 1.60E-03 | 0.1655 | Slc25a25 | 0.5373 | 1.66E-03 | 0.1724 | Zdbf2 | 0.8195 | 5.38E-05 | 6.82E-03 |  |  |  |  |
| Inmt | -0.6311 | 1.71E-03 | 0.1703 | Gm13283 | 0.6909 | 1.67E-03 | 0.1724 | C2cd4d | 0.9371 | 1.04E-04 | 0.0120 |  |  |  |  |
| Pcsk1 | 0.5781 | 1.83E-03 | 0.1789 | Clca1 | 0.5786 | 1.74E-03 | 0.1765 | Gpr3 | 0.7008 | 1.18E-04 | 0.0130 |  |  |  |  |
| Otud1 | 0.5725 | 2.03E-03 | 0.1868 | Olfr577 | -0.6981 | 1.88E-03 | 0.1816 | Paxbp1 | 0.7046 | 1.24E-04 | 0.0131 |  |  |  |  |
| Cd163 | -0.5586 | 2.23E-03 | 0.1985 | Tekt4 | -0.6663 | 1.90E-03 | 0.1816 | Retnlg | 1.1193 | 1.45E-04 | 0.0145 |  |  |  |  |
| Zfp36l2 | 0.5582 | 2.23E-03 | 0.1985 | Ptger4 | 0.5667 | 1.97E-03 | 0.1850 | Nts | 1.1122 | 1.46E-04 | 0.0145 |  |  |  |  |
| Cenpp | 0.6483 | 2.38E-03 | 0.2042 | Cd14 | 0.7055 | 2.25E-03 | 0.2035 | Baz1a | 0.8028 | 1.47E-04 | 0.0145 |  |  |  |  |
| Clk1 | 0.5498 | 2.64E-03 | 0.2232 | Det1 | -0.6143 | 2.35E-03 | 0.2035 | Cdc42ep3 | 0.6720 | 1.48E-04 | 0.0145 |  |  |  |  |
| Serpina3j | -0.5403 | 2.87E-03 | 0.2382 | Spata4 | -0.6911 | 2.54E-03 | 0.2106 | Med18 | -0.7637 | 1.54E-04 | 0.0146 |  |  |  |  |
|  |  |  |  | Fbxo33 | 0.4942 | 2.58E-03 | 0.2106 | Nsun3 | -0.8242 | 1.55E-04 | 0.0146 |  |  |  |  |
|  |  |  |  | Trhr2 | -0.5152 | 2.79E-03 | 0.2113 | Lilrb4a | 1.1014 | 1.59E-04 | 0.0147 |  |  |  |  |
|  |  |  |  | Stil | 0.8193 | 2.80E-03 | 0.2113 | Plk4 | -0.7627 | 1.74E-04 | 0.0155 |  |  |  |  |
|  |  |  |  | Tas2r139 | 0.5987 | 2.80E-03 | 0.2113 | Sinhcaf | 0.8658 | 2.06E-04 | 0.0174 |  |  |  |  |
|  |  |  |  | Olfr401 | 0.6691 | 2.83E-03 | 0.2113 | Maff | 0.9657 | 2.32E-04 | 0.0190 |  |  |  |  |
|  |  |  |  | Erbb3 | 0.5773 | 2.83E-03 | 0.2113 | Adamts9 | 0.7490 | 2.57E-04 | 0.0206 |  |  |  |  |
|  |  |  |  | Arl5b | 0.5858 | 3.04E-03 | 0.2113 | Hmgb4 | 0.8023 | 2.76E-04 | 0.0216 |  |  |  |  |
|  |  |  |  | Klf4 | 0.5976 | 3.04E-03 | 0.2113 | Cxcl2 | 0.7927 | 2.89E-04 | 0.0222 |  |  |  |  |
|  |  |  |  | Fkbp5 | -0.4809 | 3.20E-03 | 0.2196 | Gdpgp1 | -0.8443 | 3.09E-04 | 0.0234 |  |  |  |  |
|  |  |  |  | Zwilch | -0.5154 | 3.52E-03 | 0.2380 | Zfp273 | -0.6387 | 3.18E-04 | 0.0237 |  |  |  |  |
|  |  |  |  | Fam78a | -0.6184 | 3.67E-03 | 0.2414 | Mreg | -0.7207 | 3.30E-04 | 0.0243 |  |  |  |  |
|  |  |  |  |  |  |  |  | Gm9930 | 0.8930 | 3.39E-04 | 0.0245 |  |  |  |  |
|  |  |  |  |  |  |  |  | Ehd3 | -0.6637 | 3.78E-04 | 0.0266 |  |  |  |  |
|  |  |  |  |  |  |  |  | Rab2b | -0.6580 | 4.06E-04 | 0.0279 |  |  |  |  |
